# Physical key-protected one-time pad

**DOI:** 10.1038/srep03543

**Published:** 2013-12-18

**Authors:** Roarke Horstmeyer, Benjamin Judkewitz, Ivo M. Vellekoop, Sid Assawaworrarit, Changhuei Yang

**Affiliations:** 1 Departments of Electrical Engineering and Bioengineering, California Institute of Technology, Pasadena, CA 91125; 2 Biomedical Photonic Imaging Group, MIRA Institute for Biomedical Technology and Technical Medicine, University of Twente, P.O. Box 217, 7500 AE Enschede, the Netherlands

## Abstract

We describe an encrypted communication principle that forms a secure link between two parties without electronically saving either of their keys. Instead, random cryptographic bits are kept safe within the unique mesoscopic randomness of two volumetric scattering materials. We demonstrate how a shared set of patterned optical probes can generate 10 gigabits of statistically verified randomness between a pair of unique 2 mm^3^ scattering objects. This shared randomness is used to facilitate information-theoretically secure communication following a modified one-time pad protocol. Benefits of volumetric physical storage over electronic memory include the inability to probe, duplicate or selectively reset any bits without fundamentally altering the entire key space. Our ability to securely couple the randomness contained within two unique physical objects can extend to strengthen hardware required by a variety of cryptographic protocols, which is currently a critically weak link in the security pipeline of our increasingly mobile communication culture.

One-time pads are commonly acknowledged as the holy grail of cryptography^1^, but have limited application in modern ciphers. In practice, an unbreakable one-time pad (OTP) protocol requires storage of a large and random key that must remain absolutely safe against malicious attempts to copy it. As demonstrated by numerous recent database breaches, key storage is inherently vulnerable when digital electronic memory is used. Beyond surreptitious attacks via malicious software, digital electronic hardware is also susceptible to physically invasive threats^2^,^3^. Discrete reset^4^, imaging^5^ and freezing^6^ attacks can easily recover partial or entire keys. In this work, we present a new optical system and communication protocol that allows two parties to securely share gigabits of statistically random OTP keys without electronically saving any sensitive key information. Each key is derived by optically probing the randomness contained within a uniquely complex physical structure and keys are shared with OTP-strong security (i.e., eavesdropping is theoretically impossible). This novel method is extraordinarily resilient to malicious duplication attempts and is capable of securely storing communication keys at an unprecedented density. Our scheme may additionally extend to public key-based protocols, which, as photonic devices begin to solve an increasing number of integrated circuit bottlenecks, indicates volumetric scattering as a natural and efficient communication key database.

Prior optical methods of establishing encrypted two-party communication include classical spatial^7^ and temporal^8^,^9^,^10^,^11^,^12^ setups, as well as quantum key distribution^13^ (QKD). Each of these approaches, including the unconditionally secure connection offered by QKD, must electronically save its keys^14^,^15^. Developing a portable key storage medium that protects against the various threats^2^,^3^,^4^,^5^,^6^ that currently frustrate conventional electronic memory will undoubtedly improve mobile communication security^16^,^17^. Attempts at safeguarding electronic memory have applied the inherent randomness within an integrated circuit^18^,^19^,^20^, FPGA^21^ or RFID chip^22^ to implement what is often referred to as a physical unclonable function (PUF). Optical scattering-based PUF devices^23^,^24^,^25^,^26^ have also been developed to help eliminate insecure electronics, but primarily aim to create keys for terminal-based identification and authentication. All previously demonstrated PUF setups exhibit an extremely limited bit capacity – reproducible keys are on the order of kilobits.

Our work using optical scattering differs from these prior investigations in three primary regards. First, we are interested in storing keys for information-theoretically safe communication, not for identification or authentication. Second, given this new goal, we report an innovative approach for implementing an optical scattering-based communication PUF (CPUF) that can reproducibly generate gigabits, as opposed to kilobits, of statistically random keys. Third, a secure connection is only achieved after realizing some form of synchronization between the key outputs of two uniquely independent PUF devices, which constitute the encryption systems at each end of the communication line. We report a novel procedure to link the randomness enclosed by two unique physical objects without revealing any directly useful information to an adversary, which in combination with a pair of our CPUF devices allows establishment of a physically secured OTP link.

## Results

### Security expectations

**Figure 1:**
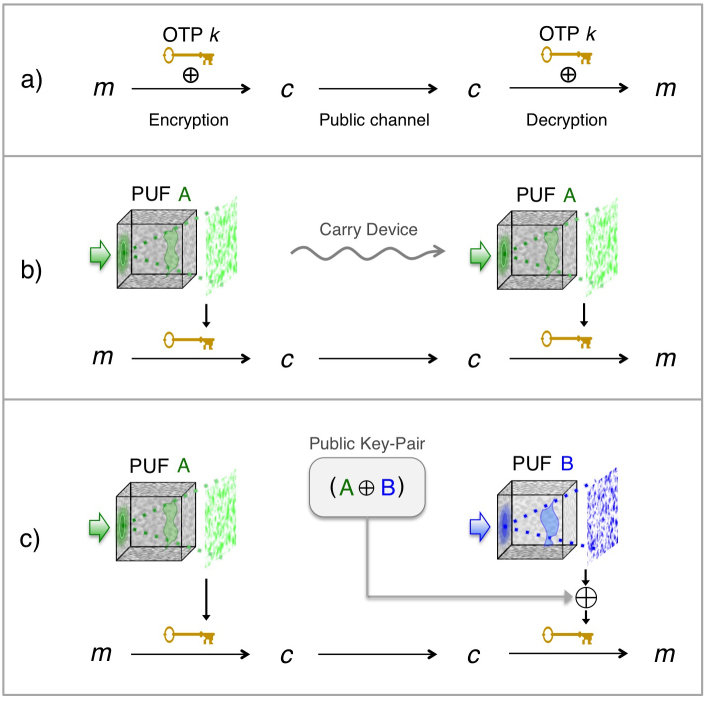
The physical one-time pad (OTP) protocol. (a) The theoretically perfect OTP mixes a binary message *m* with a random digital key *k* to create an ideally encrypted ciphertext *c* (graphics by RH). (b) A major security flaw of many communication protocols, including the OTP, is the direct ability to copy and share an electronically saved key *k*. This flaw may be addressed by storing the key within a volumetric scatterer's random structure (PUF A). Keys can be accessed with specifically shaped optical probes (green light). If the volumetric scatterer's structure is truly unique and unclonable, then physical transportation appears to be the only method of sharing keys, which is impractical. (c) The physically secure OTP protocol. The digital XOR of unique keys from PUF A and B, which itself forms an encrypted OTP ciphertext, may be publicly accessed by each party to form an information-theoretically secure link between the two uncopyable devices.

Before delving into specifics, we first briefly outline the operation and security requirements of a successfully formed CPUF channel (see Fig. 1). To communicate, two users, Alice and Bob, will first connect their personal CPUF devices over a known secure connection (e.g., by physically meeting, or by using QKD) to generate a shared random key. Once separate and mobile, they may safely exchange encrypted messages over any public channel until all shared key bits are exhausted. The basis of one-time pad cryptography stipulates that unconditional security is guaranteed only if Alice and Bob mix every message bit with an ideally random key bit without any key re-use. Throughout this paper, we assume that any key bits statistically indistinguishable from ideally random bits will sufficiently fulfill this criterion. Besides offering the OTP's unconditionally strong encryption, a “physically secure” CPUF link must meet the following requirements: first, its security must not depend upon any electronically stored data. While electronic memory may be used, no key or message fragment may be determined via a software or hardware attack targeting its contents. Second, a malicious third party (Eve) with temporary access to a CPUF must not be able to efficiently copy or model its contents. The “unclonable” nature of the CPUF's volumetric scatterer, as defined in [26], satisfies this criterion. Third, if Eve steals a device she must not be able to effectively send or receive messages. Unlike any other secure communication setup our CPUF protocol can meet these strict requirements, with a practical security limit set only by the amount of time it takes to mathematically characterize the highly complex structure of its volumetric scatterer.

### Device setup

**Figure 2:**
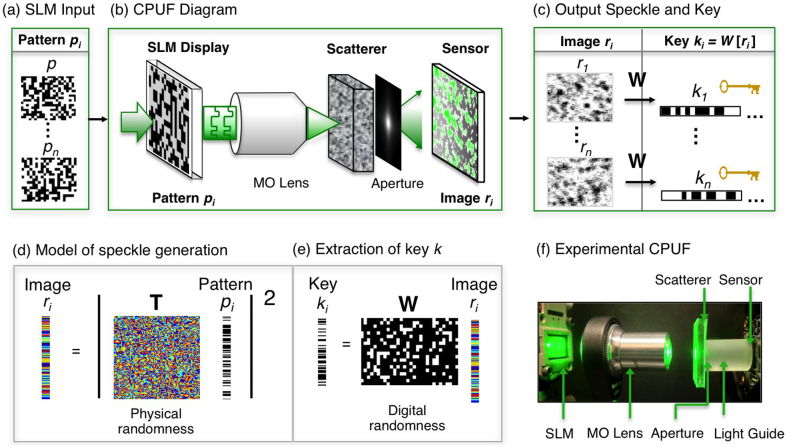
The construction and operation of a CPUF. (a) Sequentially over time, *n* random phase patterns *p_i_* are displayed on an SLM. (b) A microscope objective (MO) focuses each SLM-defined optical wavefront onto a volumetric scatterer. The scrambled light emerging from the material passes through a designed aperture before being detected by a CMOS sensor. (c) Each detected speckle image *r* is digitally transformed into a statistically random key *k* with a constant digital whitening projection *W.* (d) Optical scattering is mathematically represented by a random complex Gaussian matrix *T* and (e) digital whitening is described by a sparse binary random matrix *W*. The combination of one unique *T* and general *W* per CPUF device leads to an ideally random multi-gigabit key space that is very difficult to characterize or clone. (f) The experimental CPUF setup, including all components in (b).

We satisfy the above security expectations by storing random keys within the optical device outlined in Fig. 2. To generate a key, we first illuminate a volumetric scattering medium with a random coherent optical wavefront defined by a spatial light modulator (SLM) (Fig. 2a). An output field emerges with a profile that depends on both the SLM-defined input wavefront and the medium's random distribution and orientation of scattering particles. A designed aperture mask then shapes the output field before it propagates to an attached CMOS sensor. The mask is patterned to ensure the output speckle follows Markov statistics – an important condition for effective random key generation^27^. A combinatorially large space of possible wavelength-scale interactions enables detection of many mutually uncorrelated speckle field outputs from a set of different SLM phase profile inputs. We demonstrate how over 10 gigabits of randomness may be optically extracted from within a 2 mm^3^ volume. Optically addressing the PUF scatterer with an array of SLM pixels is the primary experimental insight enabling extraction of multiple orders of magnitude more bits than any prior work.

We can mathematically describe this large amount of extractable physical randomness by representing optical scattering with a random complex Gaussian transmission matrix^28^ ***T*** (Fig. 2d). The output scattered field created by displaying the *i^th^* random SLM phase pattern ***p_i_*** may be described by ***u_i_**** = ****T**** · ****p_i_****.* After the sensor detects the output field's intensity ***r_i_*** ≡ |***u_i_***|^2^, a fixed whitening and noise removal operation ***W*** is applied to the speckle pattern to create a statistically verified random and repeatable key ***k_i_*** (Fig. 2e). The SLM pattern ***p_i_*** and output key ***k_i_*** are thus connected by, 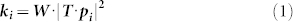
The projection of optical field ***p_i_*** into the random matrix ***T***, uniquely defined by the volumetric scatterer within each CPUF device, imparts key ***k_i_*** with its unclonable security.

### Physically secure protocol

**Figure 3:**
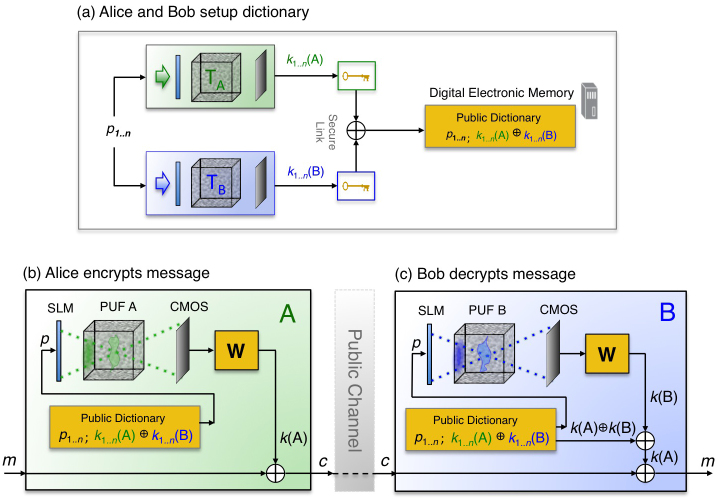
Ideally secure CPUF communication protocol. (a) During setup, Alice and Bob securely connect their two devices and each generate *n* CPUF keys *k*_1*..n*_(*A*) and *k*_1*..n*_(*B*) using the same *n* input SLM patterns *p*_1.*.n*_. Each key-mixture *k_i_*(*A*) 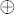 *k_i_*(*B*) is saved in a digital electronic dictionary that is assumed public, along with its corresponding SLM pattern *p_i_*. (b) At a later time *t_c_*, Alice may send Bob an unconditionally secure ciphertext *c* by selecting a pattern *p*, re-creating key *k*(*A*), and then XORing this key with her message *m*. The public dictionary can be saved locally on each device without any sacrifice to security. (c) Bob decrypts the received ciphertext. He uses *p* to both re-generate key *k*(*B*) and to find the public dictionary's corresponding key-mixture. The XOR of *c* with *k*(*B*) and the key-mixture reveals *m*. No secret key is ever digitally stored, obfuscating any copy attack.

A set of *n* random keys ***k_1..n_***(*A*) generated by Alice's CPUF, along with a corresponding key set ***k_1..n_***(*B*) generated by Bob's CPUF, enable physically secure OTP communication with the assistance of a digitally saved public dictionary (Fig. 3). As described above, Alice and Bob begin by establishing a secure connection between their two devices. While connected, they sequentially probe their scatterers with the same set of *n* random SLM phase patterns ***p_1..n_***, respectively detecting key sets ***k_1..n_***(*A*) and ***k_1..n_***(*B*) following equation (1) (Fig. 3a). Key sets ***k_1..n_***(*A*) and ***k_1..n_***(*B*) reflect each device's unique transmission matrix ***T_A_*** and ***T_B_*** but remain synchronized through Alice and Bob's shared use of SLM set ***p_1..n_***. Without leaving any digital trace of an individual key, Alice and Bob populate a public dictionary with each SLM pattern ***p_i_*** paired with the digital XOR of the two keys it generates, ***k_i_***(*A*) 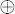 ***k_i_***(*B*), for 1 ≤ *i* ≤ *n*. An eavesdropper will gain no information about an individual key from this saved XOR “key-mixture”, since it takes the form of a secure OTP ciphertext.

Once mobile at a later time *t_c_*, Alice may send Bob an encrypted message ***m*** by first randomly selecting an unused pattern ***p_i_*** from the public dictionary to re-create key ***k_i_***(*A*) (Fig. 3b). Then, Alice may use this key to create and send an XOR-encrypted ciphertext ***c***, where ***c*** = ***k_i_***(*A*) 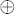 ***m*** (here we assume ***k_i_***(*A*) and ***m*** are the same length – longer messages are encrypted by concatenating multiple keys). To complete the protocol, Alice must also send Bob the index *i* of the SLM pattern ***p_i_*** she displayed, which need not be encrypted.

Bob decrypts Alice's ciphertext using both his CPUF as well as the public dictionary (Fig. 3c). He displays ***p_i_*** to optically regenerate key ***k_i_***(*B*), and accesses dictionary entry *i* to obtain the corresponding key-mixture [***k_i_***(*A*) 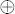 ***k_i_***(*B*)]. The decoded message is then obtained by an XOR of these two sequences with the received ciphertext: 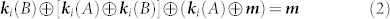
The total number of secure bits *N* that Alice and Bob may share is proportional to the product of the number of saved key-mixtures *n* and the number of bits within each key |***k***|. Factors that limit *N* include display and sensor resolution, scatterer size, and allowed setup time (Supplementary Text A).

The security of the above protocol relies upon the CPUF key sets following what Shannon defines a purely random process^1^. Possible deviations from pure randomness fall into three categories: correlated bits within the same key, correlations between keys, and the introduction of noise between keys generated at time *t_0_* and at time *t_c_*. The sparse projection operator ***W*** overcomes such deviations to create keys that asymptotically approach information-theoretic security by sacrificing an increasing number of available encryption bits^29^. In practice, ***W***'s bit reduction factor is selected such that each CPUF key set ***k_1..n_***, viewed as one multi-gigabit random sequence, passes all tests contained within the Diehard^30^ and National Institute of Science and Technology (NIST)^31^ statistical random number generator test suites. These two test packages are commonly accepted as the standards for random sequence certification. While often used to ensure the suitability of pseudo-random generators for nearly all cryptographic applications, they also effectively verify physical randomness generation^11^,^12^. Performance statistics of the NIST test applied to an example 10-gigabit CPUF key set are presented in Table 1. Diehard test results for the same key set are included in Supplementary Text D. Finally, context-tree weighting (CTW) compression offers a third independent strategy to thoroughly verify the randomness of PUF-produced keys^32^. Applying the same CTW algorithm in [32] to an example 1-gigabit CPUF key set generates a completely uncompressed output (i.e., 0 bits are redundant, indicating the average entropy-per-bit is 1). Supplementary Text D offers further details regarding the above three statistical tests and the specific parameters used to extract each multi-gigabit key set.

### Experiment

The CPUF devices used in experiment each contain a 2 megapixel transmissive phase SLM imaged onto opal-diffusing glass, serving as our highly random scatterer. A 2 cm light guide (to increase the average detected speckle size) connects the scatterer to a 4.9 megapixel CMOS detector. During public dictionary setup, we display *n* = 5,000 different random phase patterns ***p*** to generate 174.4 gigabits of raw speckle data from two CPUFs, which is reduced via the sparse matrix operator ***W*** to the *N* = 10 gigabits of randomness statistically verified in Table 1. The approximate theoretical limit of 150 gigabits of randomness per CPUF link may be achieved using a thicker volumetric scatterer, currently limited to 0.5 mm for optical stability purposes. Improved-resolution SLM and CMOS arrays may allow random bit densities to eventually reach 1 terabit/mm^3^. These two bit density limits are both derived in Supplementary Text A.

**Figure 4:**
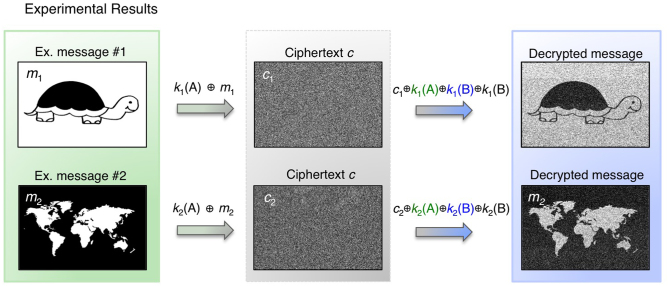
Experimental message transmission. Example messages *m_1_* and *m_2_* are sent between two synchronized CPUF devices 24 hours after dictionary setup (each message image created using Paintbrush). Each message is encrypted to and decrypted from a statistically random ciphertext *c*. Here, error correction by repetition coding (code rate = 0.025) helps reduce noise during decryption.

Experimental communication between two CPUFs following our physically secure OTP protocol is demonstrated *t_c_* = 24 hours after public dictionary setup in Fig. 4. Due to the slight drift of scatterers, message noise is introduced upon decryption. For example, direct application of equation (2) to decrypt a standard 40-megabit ciphertext after a 24-hour delay produces a message with a 0.4 bit-error-rate (BER). Borrowing concepts from the area of fuzzy commitment^33^, we can use error correction codes to reduce this noise without impacting protocol security. However, the application of error correction will also reduce the total number of securely transmittable bits by a fixed fraction, known as the code rate. For example, a simple error correction strategy based on a code-offset construction^33^ with repetition coding (code rate = 0.025) improves the average message BER to 0.21. A more computationally demanding BCH coding strategy^34^ (code rate = 0.035) improves this average BER to 0.17. We can estimate our CPUF setup's maximum achievable code rate, independent of a specific error-correction strategy, using the CTW algorithm mentioned above. Following [32], an independent mutual information estimate of our tested 40-megabit encryption and decryption keys sets our code rate upper bound at 0.18, indicating that 18% of our 10-gigabit key sets may lead to noise-free secure communication. Future work will attempt to reduce inter-key noise and identify an optimal error correction strategy to reach the code rate upper bound without leaking any sensitive information.

## Discussion

By linking two physical reservoirs of randomness without relying upon any secure electronic storage, our CPUF principle fundamentally transforms how two parties can establish an ideally protected communication link. While the unconditional algorithmic security of the proposed protocol is well understood, the CPUF device's physical security is slightly harder to systematically analyze. To begin, given an appropriate average scattering particle size and density, the odds of creating or finding two unique CPUF devices that share an overlapping key sequence are astronomically low (approaching 2^−|*k*|^ for an ideal scattering volume). Furthermore, attempts to maliciously determine keys through physically probing a CPUF's unclonable^26^ scattering volume will irreversibly alter its optical response and render its protocol ineffective^24^. In addition to utilizing scatterer storage, our protocol also relies upon a limited number of digital electronic operations that may be subject to physical scrutiny. Since no permanent digital memory is used (except to hold the unconditionally secure key-mixture), the rate of any electronic attack is fundamentally limited by the optical transfer speed between the scatterer and an accurate CMOS sensor detection, which we define as the maximum key generation rate available to Alice and Bob.

Assuming an eavesdropper Eve can undetectably steal a device, the above limitations set our current CPUF protocol's functional lifetime upper bound at approximately 50 hours. This lifetime is fixed by the approximate time an eavesdropper will require to mathematically characterize the random structure of an ideally operating CPUF at the current capture rate of ~1.5 seconds per key. Faster capture is not possible due to induced scatterer heating (see Supplementary Text F). This functional lifetime scales linearly with the device's achievable key space *N*, which may be easily increased by at least an order of magnitude with a corresponding improvement in sensor and SLM resolution. Scatterer heating may likewise be used advantageously to reset or destroy each CPUF, which makes it impossible for Eve to recover any previously communicated message.

Through appropriate channel monitoring, it is also possible for Eve to use a stolen device to directly recover previously communicated messages. To prevent such an attack, Alice and Bob may adopt a public-key protocol to computationally protect their shared screen patterns from quick readout. As detailed in Supplementary Text F, simply replacing each SLM pattern ***p_i_*** in their shared dictionary with a derived private key PK[***p_i_***] offers a fundamental advantage: given Alice's device, Eve must either contact Bob to determine each SLM pattern PK[***p_i_***] to use as a probe, or attempt to computationally recover each pattern PK[***p_i_***] by overcoming the discrete logarithm problem. This simple modification 1) compels Eve to successfully decrypt *each* SLM pattern before being able to recover the previously transmitted message associated with it, and 2) forces Eve to measure the full scattering matrix ***T*** to determine *any* future communicated message. While no longer tied to unconditional security, protecting the CPUF's multi-megabit probe functions ***p*** with traditional cryptographic protocols is a unique feature of our device's architecture. We expect this fusion of practical digital security principles with physical storage (i.e., a form of multi-factor authentication) will be the subject of much future work, and will allow the CPUF to adapt to meet the challenges provoked by any inevitable side-channel attacks.

In conclusion, the demonstrated CPUF system applies optical scattering to access billions of random bits stored within an unclonable volumetric structure. Information-theoretically secure communication is achieved using a modified OTP protocol. While such ideal protection is a good starting point for any new cryptographic mechanism, physical CPUF keys can also extend to public-key exchange, which eliminates an initial secure connection requirement but fails to guarantee OTP-strong security (see Supplementary Text F). Compared with a large, electronically saved one-time pad, the CPUF's key is extremely challenging to copy or model and can easily scale to provide terabits of repeatable randomness within a small volume. Embedding the device's digital electronics within its volumetric scattering material will further impede any attempted copy or probe attack. With additional study, we hope the convenient properties of optical scattering can solve enough of the OTP's practical shortcomings to rejuvenate interest in its unbreakable security, even in the presence of infinite computing resources.

## Methods

### Methods summary

One CPUF device uses a spatially filtered and collimated solid-state 532 nm CW laser (Spectra-Physics, Excelsior Scientific 200 mW) to illuminate a transmissive SLM (1920 × 1080 pixel, 1 × 1.6 cm Epson HDTV LCD, BBS Bildsysteme). The SLM is operated as a phase modulator (without a second polarizer). The scatterer-detector CPUF segment is composed of four main components that are fastened together using an epoxy to minimize any movement with respect to one another. First, the base of the CPUF is a 2.2 μm, 2592 × 1944 CMOS pixel array (The Imaging Source, Micron CMOS MT9P031) with USB readout to a desktop computer. Second, a glass light guide (1.24 cm Quartz disk, Mcmaster-Carr 1357T62) is fixed to the surface of the CMOS protective glass. Third, a custom-printed amplitude-modulating mask (Kodak LVT-exposed on film at 2032 dpi, Bowhaus Printing, 5 mm × 5 mm) is attached to the glass light guide to serve as the speckle-shaping aperture. The aperture size is designed to be approximately 1 mm across, ensuring the average speckle size extends across 5 sensor pixels, enhancing speckle image stability. The apodizing mask follows a 2D-separable Cauchy distribution to ensure the speckle exhibits the required properties of a Markov random process (see Supplementary Text B). 99% of the mask's transmitted light is contained within its central 1.2 mm^2^ area. Fourth, the volumetric scattering material – opal diffusing glass with 0.5 mm scattering volume thickness (Edmund Optics NT46-645) – is fixed above the aperture mask.

### Key acquisition and processing

A low laser power (~0.2 μW) was used to illuminate each CPUF, preventing speckle pattern decorrelation from material heating but requiring a ~1.5 second image exposure time. Image capture and SLM screen control were driven through a Matlab interface. After capture, the speckle was transformed into a 1D vector and whitened into a key via multiplication with a matrix ***W*** (detailed in Supplementary Text C). One large sparse ***W*** is stored locally on a desktop computer for use by two CPUF devices, along with the public dictionary containing the shared set of *n* binary random SLM patterns and the XOR key-mixtures from connection setup. ***W*** need not be unique for each CPUF device. Furthermore, its contents may be known publically without any loss of protocol security. Each binary SLM pattern was selected uniformly at random from [0, π] to minimize the probability of key collision. Communication was experimentally achieved between two similarly constructed CPUF devices by populating a public dictionary, waiting 24 hours, and then using the same optical setup to execute the protocol outlined in Fig. 3. Specific encrypt-decrypt parameters for the transmitted messages in Fig. 4 are outlined in Supplementary Text C, where each message contains 0.4 megabits after applying error correction, as detailed in Supplementary Text E.

## Author Contributions

R.H., B.J. and I.V. and C.Y. conceived and developed the initial idea together. I.V. conceived the final protocol. R.H. designed the experiment, built the setup, and collected the data. R.H. and S.A. performed data and security analysis. All authors collectively wrote the manuscript.

## Supplementary Material

Supplementary Information Supplementary Text

## Figures and Tables

**Table 1:** Example NIST statistical randomness test performance. NIST statistical randomness test package performance of a typical 10-gigabit CPUF key set from experiment, split into 10,000 unique 1 megabit sequences following a common procedure^11^,^12^. For ‘success’ using 10,000 samples of 10^6^ bit sequences and significance level α = 0.01, the p-value (uniformity of p-values) should be larger than 0.0001 and the minimum pass rate is 0.987015. Tests that produce multiple p-values and proportions are denoted by a (^+^), followed by the number of different test values generated in parenthesis. The table displays the lowest (i.e., worst-case) generated p-values and proportions in the set

Statistical Test	p-value^+^	Proportion	Pass/Fail
Frequency	0.128	0.9895	Pass
Block Frequency	0.053	0.9925	Pass
Cumulative Sums	0.388^+^ (2)	0.9897	Pass
Runs	0.760	0.9908	Pass
Longest Run	0.327	0.9899	Pass
Rank	0.028	0.9892	Pass
FFT	0.021	0.9874	Pass
Non-overlapping Template	0.003^+^ (147)	0.9894	Pass
Overlapping Template	0.002	0.9879	Pass
Universal	0.226	0.9886	Pass
Approximate Entropy	0.156	0.9901	Pass
Random Excursions	0.163^+^ (8)	0.9873	Pass
Random Excursions Variant	0.006^+^ (18)	0.9902	Pass
Serial	0.031^+^ (2)	0.9896	Pass
Linear Complexity	0.887	0.9889	Pass
