# Supplementary material for: Physical key-protected one-time pad
Source: Sci Rep. 2013 Dec 18;3:3543. doi: 10.1038/srep03543 (PMC3866593; doi:10.1038/srep03543)
Supplement: Supplementary Information — Supplementary Text [file srep03543-s1.pdf]

Physical key-protected one-time pad: Supplementary information

R. Horstmeyer, B. Judkewitz, I. Vellekoop, S. Assawaworrarit and C. Yang

Supplementary Text A: Expected number of random bits per device

The number of useful random bits N extractable from an ideal CPUF device over its lifetime is limited by two physical phenomena. First, correlations between the pixels of each speckle image (caused by a finite average speckle size) limit the size of each random key. Second, correlations across the set of all possible speckle images place an upper limit on the number of uncorrelated random keys that each CPUF can produce. The whitening operator $\boldsymbol{W}$ removes correlations by decreasing the number of output random bits N. $\boldsymbol{W}$ allows the N-long output to asymptotically approach a completely random sequence as N becomes small, as detailed in Supplementary Text D. Here, we find an approximate upper bound on the total number of random bits that we can expect $\boldsymbol{W}$ to extract from one CPUF device. This upper bound, derived from experimental measurements, is based on the product of the minimum number of random bits per image β and the total number of uncorrelated images n per device: $N \leq \beta \cdot n$. Equality is achieved assuming the correlations contained within each speckle image do not vary between images.

1. Number of random bits per speckle image β

A rough estimate of the number of random bits contained within each speckle image β is found by considering the entropy rate of a discretely detected speckle intensity pattern. The following calculations are based upon common assumptions regarding speckle detected a finite distance away from an ideal scattering surface[35]. We discuss the optical setup assuming a 1D geometry for simplicity, with direct extension to 2D. We assume a coherent, polarized, monochromatic field with many de-phased contributions as the field source at the back surface of the volumetric scatterer, which propagates to form a random speckle field $u(x)$ following circularly symmetric complex Gaussian statistics at the CMOS detector plane. The detector discretely samples and measures the field's intensity $|u(x)|^2$ at g_x pixels each of width δ_x, across which we assume the speckle field to follow spatially stationary statistics (i.e., the speckle statistics are spatially uniform). Each pixel in the detector exhibits a finite bit depth b (typically 8 bits), generating a detected intensity magnitude discretized into 2^b bins.

For a direct random bit estimate, we initially assume that each speckle exhibits a finite correlation within a particular range d, fixed by the average size of one speckle, and is zero elsewhere. This is equivalent to assuming the speckle exhibits a *rect* autocorrelation function of width d, which closely approximates the typical $sinc^2$ correlation function of speckle generated through an open aperture, given a small average speckle size. Such an approximation is found accurate in many practical systems[35], and is displayed in Supplementary Figure S1(a) along with an experimental CPUF speckle autocorrelation using a circular aperture geometry (autocorrelation width d = 6 pixels). See Supplementary Figure S2 for a diagram of the setup with variables of interest labeled. A finite correlation estimate offers the intuitive picture of g_x total sensor pixels measuring g_x/d independent speckles, with each independent speckle extending across d pixels. A *rect* autocorrelation approximation allows us to replace the correlated speckle sequence $|u(x)|^2$ containing p_x discrete measurements with a shortened vector $\boldsymbol{v}$ containing g_x/d random variables, which we assume are uncorrelated given a sufficiently large d.

A sequence of dim($\boldsymbol{v}$) biased (i.e., imperfect) random variables can be converted into a shorter sequence of β independent, unbiased random bits using a procedure known as randomness

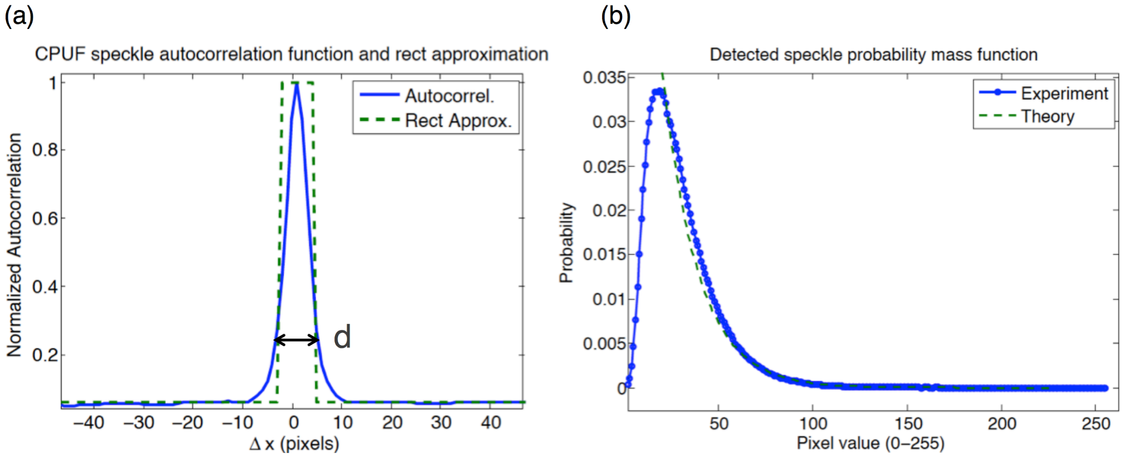

Supplementary Figure S1 | CPUF characterization data. Experimental data used to derive an estimated upper bound on the number of random bits per detected image. (**a**) Speckle autocorrelation function obtained from our CPUF device. To derive a direct entropy upper bound, we approximate the experimental curve with the plotted *rect* function of width d. (**b**) The probability mass function of speckle detected by the CPUF device, compared with a theoretical exponential curve[35]. Deviations from the exponential curve at lower pixel values are caused by the detection of depolarized light at the scatterer back surface.

extraction[36]. We will use dim(·) to denote the dimension, or number of random bits, within a binary vector. The efficiency of extraction is bounded by the entropy rate $H'(\boldsymbol{v})$ of the random process $\boldsymbol{v}$. In other words, the number of unbiased random bits β we may obtain is limited by our random process's entropy. Assuming $\boldsymbol{v}$ follows spatially stationary statistics, we may calculate β with,

$$\beta = (g_x/d)H(\boldsymbol{v}) = -(g_x/d)\sum_{i=0}^{2^b-1} P(v_i)\log_2 P(v_i), \tag{3}$$

where $P(v_i)$ is the probability of one pixel taking on value v_i, the sum represents the per-pixel entropy $H(\boldsymbol{v})$, and the sum is performed over 2^b detectable values. Here, per-pixel entropy may replace entropy rate assuming an identically distributed process across the sensor, which follows from our assumption of spatial stationarity[37]. An accurate estimate of the probability mass function $P(\boldsymbol{v})$ is achieved through an experimentally generated histogram of detected values, shown in Supplementary Figure S1(b). Based on this experimentally determined mass function, the entropy rate of the CPUF's speckle intensity is $H(\boldsymbol{v})$ = 5.78 +/- 0.07, where variations are associated with a slightly fluctuating image histogram over many experiments. Assuming a 2D separable geometry with a sensor containing 2592 x 1944 pixels and estimating the average speckle correlation width to extend over d = 6 pixels in both dimensions, we arrive at an estimated β = 1.16 x 10^6 bits of randomness per detected image, following equation (3). We note this experimental procedure may be applied to any CPUF setup to arrive at a device-specific maximum random bit estimate. For example, scaling the current system to use a commercially available 40 megapixel sensor will result in each key containing close to 10 million random bits.

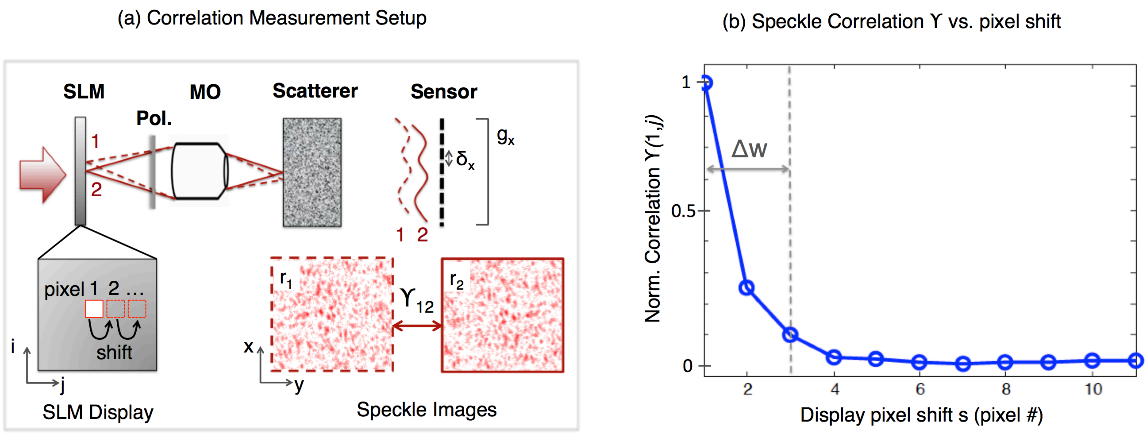

Supplementary Figure S2 | Removing scattering's inter-mode correlations. (**a**) Experimental setup to measure the correlation between adjacent speckle modes. Sequentially turning on neighboring amplitude-modulating SLM pixels and recording speckle images allows calculation of correlation function γ. (**b**) A plot of correlation parameter γ from equation (4) for the tested CPUF. From this plot, it is clear that additional correlations exist between up to 4-6 pixels. We minimize these correlations by grouping SLM pixels into 4 x 4 block segments.

2. Number of uncorrelated speckle images per device n

The number of uncorrelated speckle images per CPUF device n may also be estimated through experimentally measured quantities. An upper bound on the number of independent speckle images needed to characterize a PUF device has been theoretically derived before in ([23]) and via information theoretical arguments in ([38]), which focuses on short keys used for identification. The upper bounds offered in this prior work assume various idealities that do not exist in our CPUF device. We instead derive a more accurate independent image estimate tailored to our CPUF's geometry, resolution and scattering material specifics through experimentally measured quantities, which is then compared to prior theoretical estimates.

First, we examine the transmission matrix $\boldsymbol{T}$ to arrive at a loose upper bound on the number of independent speckle images per device, n. Then, we refine n's estimate using experimental measurements. We begin with the ideal assumptions that $\boldsymbol{T}$ is an l x l random unitary matrix where l is the number of scattering modes[39], and that the SLM and sensor used with the setup each contain l pixels. A unitary approximation generally holds for highly scattering medium with many open channels (i.e., l is large). Deviations from this ideal condition are considered below. A unitary $\boldsymbol{T}$ guarantees the rank of $\boldsymbol{T}$ is l, and that its inverse exists as $\boldsymbol{T}^*$, the complex conjugate of $\boldsymbol{T}$. In this case, we prove that l uncorrelated speckle images exist, and thus the upper bound on the ideal number of uncorrelated keys that can be extracted from $\boldsymbol{T}$ is $n = l$, as follows. Since the inverse of a unitary $\boldsymbol{T}$ exists and $\boldsymbol{T}$ is full rank, we can find a unique $\boldsymbol{p}$ that satisfies the equation $\boldsymbol{u} = \boldsymbol{T}\cdot\boldsymbol{p}$ for any *real, positive* vector $\boldsymbol{u}$ by solving $\boldsymbol{p} = \boldsymbol{T}^*\cdot\boldsymbol{u}$. Thus, $\boldsymbol{u}$ can be replaced with the quantity $\boldsymbol{r} = |\boldsymbol{u}|^2$ without loss of generality. Given that $\boldsymbol{p} = \boldsymbol{T}^*\cdot\boldsymbol{r}$ for any intensity vector $\boldsymbol{r}$, we then see that an l x l, rank l matrix $\boldsymbol{P}$ can be constructed from a set of l orthogonal intensity column vectors combined into an l x l matrix $\boldsymbol{R}$ by solving $\boldsymbol{P} = \boldsymbol{T}^*\cdot|\boldsymbol{U}|^2 = \boldsymbol{T}^*\cdot\boldsymbol{R}$. $\boldsymbol{P}$'s full rank is trivially verified when $\boldsymbol{R} = \boldsymbol{I}$, the identity matrix. The existence of rank-l speckle intensity matrices $\boldsymbol{R}$

(real, positive) and $\boldsymbol{P}$ that satisfy the scattering equation thus indicates $\boldsymbol{T}$ can generate l uncorrelated speckle intensity images. In other words, the number of scattering modes in $\boldsymbol{T}$ dictates the number of uncorrelated keys it can generate.

Assuming an ideal scatterer, the number of scattering modes l is proportional to the number of wavelength-sized spots that fit within the area A_0 of the optical probe illuminating the scattering material through $l = 2\pi c_0 A_0 / \lambda^2$, where c_0 is a constant of order unity and we assume access to both polarizations[40]. From a calculated focal area of $A_0 = 1.6\text{mm}^2$ for the current setup's 10x objective lens imaging a 1.0 x 1.6cm display, we arrive at $l = 3.55 \times 10^7$ ideally addressable optical modes. This large number of scattering channels cannot be probed by the current CPUF's SLM display due to its limited resolution (2.09 megapixels). Thus, the rough upper bound on the uncorrelated key count can be reduced simply to $n = 2.09 \times 10^6$, the number of possible orthogonal probes available on the SLM. Future CPUF designs may better approximate the above ideal upper bound using an SLM display containing many more pixels.

The accuracy of the estimate of n is further increased by experimentally measuring deviations of CPUF scattering from the ideal operation of $\boldsymbol{T}$, which is not perfectly unitary, in practice. Three specific forms of correlation are known to exist within the transmission matrix[41]. We experimentally account for the most dominant first-order correlation effect contained by $\boldsymbol{T}$ as follows. First, the SLM display is used in amplitude transmission mode by inserting an appropriately oriented polarizer between it and the scatterer. Only one SLM pixel at location (i, j) is turned on (i.e., is made optically transparent) with all other pixels off, and a speckle image is recorded. We repeat this process, turning on SLM pixel $(i+s, j)$ and recording a speckle image, for $s = (1, \ldots, 20)$. The similarity between the initial and s^{th} speckle image r_s is calculated through a mean-subtracted overlap function γ:

$$\gamma_k = \sum_{a=1}^{g_x} (r_1(a) - \overline{r_1})(r_s(a) - \overline{r_s}), \tag{4}$$

where the sum is performed over each image's sensor pixels and is considered only along one sensor dimension. The results of such an experiment are in Supplementary Figure S2(b), where the presence of additional correlations extending out to $\Delta w = 4$ SLM pixels are clear. These correlations appear to be an indication of the scattering memory-angle effect[42], but may also suggest a partially complete scattering process (i.e., $\boldsymbol{T}$ is not full-rank). We reduce these correlations, leaving <10% of γ's significance remaining, by grouping all SLM pixels into 4 x 4 sets. This grouping further reduces the number of SLM degrees of freedom n by a factor of 16 to $n = 1.3 \times 10^5$.

Comparing our final experimental bound on n to two prior theoretical approaches, we first find that the memory-angle method in ([23]) leads to 1.8×10^7 probable modes assuming a 10x objective and 0.5mm scatterer thickness. Second, comparing our results to the simple upper bound in ([38]) shows that our derived number of modes l is exactly half their upper bound, as polarization is not taken into account. Both values are close to our derived bound of $l = 3.55 \times 10^7$, and both are greater than the current number of SLM pixels ($n = 2.09 \times 10^6$).

3. Total random bits per CPUF device N:

A tight upper bound on the total number of uncorrelated random bits per experimentally tested CPUF device N is given by the product of the number of unbiased bits per image β in Supplementary section A.1 and the number of uncorrelated images per device n in Supplementary

section A.2 as the following:

$$N \leq \beta \cdot n = (1.16 \text{ x } 10^6 \text{ bits/image})(1.3 \text{ x } 10^5 \text{ images/device}) = 1.51 \text{ x } 10^{11} \text{ bits.} \quad (5)$$

Equation (5)'s upper bound easily scales to tens of terabits using commercially available SLM displays and CMOS sensors offering improved resolution.

In a practical encryption scheme, a limited database setup time may enforce a tighter bound on n than suggested by equation (5). Currently, equation (5)'s upper bound requires over one day of CPUF setup time before a complete database of 10^5 images is populated (28 hours at an approximate capture rate of 1 second per image). An approximate one-second delay between captured images is required to prevent scatterer heating, which causes keys to decorrelate over time. Future setups may attempt to achieve a practical upper bound by increasing the CPUF setup speed until the scatterer significantly decorrelates. Given the ability to avoid decorrelation, N's upper bound can be detected by increasing n until the entire OTP sequence begins to fail statistical randomness tests due to introduced correlations. However, an exact statistical upper bound is difficult to determine for large n due to computational limitations. For example, the NIST randomness test applied to an n = 5000 raw speckle key set requires approximately 30 hours on a modern processor (2.5GHz, 16 GB RAM), scaling linearly until memory is exhausted.

4. Ideal upper bound, expected number of random bits

The derivation in the above sections 1-3 incorporates our current setup's non-ideal SLM and CMOS sensor pixel sizes to reach an accurate upper bound on the number of random bits per tested CPUF device. Neglecting these large pixel sizes leads to a much greater *ideal* number of random bits per device (i.e., an upper bound estimate for an optimal CPUF setup). For simplicity, we determine this idealized upper bound per 1 mm^3 of scatterer volume. Four assumptions lead to an efficient calculation. First, we safely assume a 1 mm scatterer thickness can generate a fully random T matrix[39]. Second, we pessimistically assume that such a cubic volumetric scatterer contains $l \sim 10^6$ modes following the previously noted[40] equation, $l = 2\pi c_0 A_0 / \lambda^2$. Third, we assume access to an idealized SLM than can efficiently access all l of these scattering modes. Fourth, we assume that an ideal detector placed at the back surface of the 1 mm^2 scattering surface can obtain $(1\text{mm}/\lambda)^2$ independent measurements. This corresponds to 4 x 10^6 pixels per 1 mm^2 of detector area with an illumination wavelength of λ = 500nm. The product of the number of independent modes with the number of measurements per mode yields 4 x 10^{12} measurements following an independent, biased process. Binary sampling to ensure an unbiased binary process leads us to a final approximation of over 1 terabit of randomness per 1 mm^3 scattering volume for an idealized CPUF setup. This large amount of ideal randomness indicates that future devices should be able to easily improve upon the current number of derived (1.5 x 10^{11}) and experimentally achieved (10^{10}) random bits. Furthermore, this large space of randomness will scale linearly with scatterer area for a sufficiently thick volume, given the above ideal conditions.

Supplementary Text B: Patterned apodizing mask

The CPUF's amplitude-modulating apodizing mask serves three main purposes, two of which require its specific shape (Supplementary Figure S3). First, the mask provides control over the size and shape of the exit pupil at the back surface of the scattering material. Assuming for simplicity that the mask is a circular aperture of radius w_a, the average speckle size d at the sensor is linked to w_a through the simple relationship $d = \lambda \cdot z \cdot w_a^{-1}$, where z is the distance between the

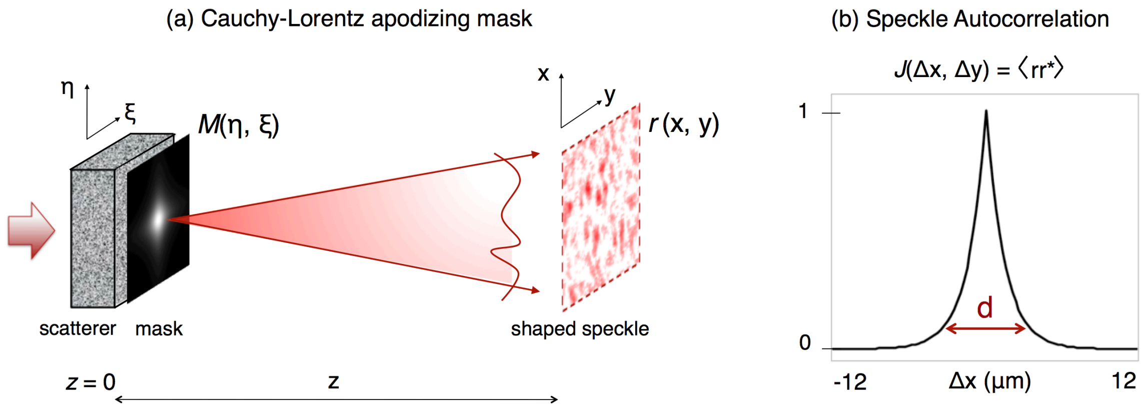

Supplementary Figure S3 | A designed apodizing mask guarantees speckle randomness. (**a**) A Cauchy-Lorentz amplitude-modulating mask $M(\eta, \xi)$ ensures the detected speckle obeys a hidden Markov process across space at the detector plane, required by the whitening operator W to ensure efficient randomness extraction. (**b**) The Cauchy-Lorentz aperture leads to an exponentially correlated speckle field following a first-order Markov process, which in turn causes the intensity to follow a hidden Markov process, as demonstrated in ([27]).

scatterer and sensor[35]. The average speckle size d should be carefully chosen to extend over several sensor pixels. A large d offers more setup stability, but does so at the expense of sacrificing the number of extractable random bits per key.

Second, a more complete description of the shape of an average speckle is arrived at when an arbitrarily-shaped aperture distribution $M(\eta, \xi)$ is considered. The autocorrelation function of the speckle field u at the sensor plane is related to M via a Fourier transform relationship[35]:

$$J(\Delta x, \Delta y) = \langle u(x,y)u^*(x,y)\rangle_{\Delta x, \Delta y} = (k/\lambda^2 z^2)\mathcal{F}[|M(\eta,\xi)|^2] \quad (6)$$

where a constant phase factor is neglected, k is the wavenumber and $\mathcal{F}$ represents a Fourier transform operation. The speckle intensity's autocorrelation J_I is related to equation (6)'s field autocorrelation J through the simple relationship, $J_I = 1 + |J|^2$. The average shape of the detected speckle, defined by its intensity autocorrelation, may thus be controlled with a designed aperture mask function $M(\eta, \xi)$.

One choice of mask function guarantees that speckle processed by the whitening operator $\boldsymbol{W}$ converges to a uniformly random sequence. It is shown in ([29]) that $\boldsymbol{W}$, based on projecting our speckle vector r into a sparse subspace with lower dimension, converges in statistical distance to an ideal random sequence when r follows a Hidden Markov Process (HMP). It is demonstrated in ([27]) that an apodizing mask $M(\eta, \xi)$ with an attenuation profile following a 2D-separable Cauchy-Lorentz distribution generates a complex speckle *field* that follows a first-order Markov process at the sensor.

This condition also guarantees the CPUF's detected speckle *intensity* pattern follows a 2D HMP across space, as the field and intensity are connected by an underlying state-observation

relationship. Specifically, the HMP's discrete state space is comprised of the field's possible complex values and its observation space is the discrete set of 2^b detectable speckle intensity values. Its emission matrix contains the conditional probably of observing intensity value $r(v_i)$ from any complex field value $u(v_i)$, and all matrix entries will all be zero except those which obey the deterministic relationship, $r(v_i) = |u(v_i)|^2$. In summary, a second application of an included apodizing mask M, if it follows a Cauchy-Lorentz distribution, is to create HMP speckle, which is proven to approach a truly random sequence of bits almost surely after digital whitening.

Third, a Cauchy-Lorentz apodizing mask serves to increase the detected speckle's entropy, and thus maximizes its number of extractable random bits for a fixed speckle size. A detailed support of this claim is also presented in ([27]). Here, we add three clarifying points. First, while the proof of random bit maximization in ([27]) is an exact solution for a detected complex field, extension to a detected intensity is direct following Theorem 9.6.5 in ([37]), which may lead to a maximized entropy upper-bound. Second, by considering entropy for a fixed dynamic range, we implicitly assume that any photons lost to blocked light at the aperture are made up for by increasing the camera's shutter time. And third, this entropy maximization is for a field with fixed average speckle size d, with the relative entropy gain being low for small values of d, as in the case of the experimental CPUF setup where d only extends across approximately 6 sensor pixels.

Supplementary Text C: Noise reduction and digital whitening

Information-theoretically secure communication is achieved with an OTP encryption key that follows an ideally random sequence. A vector of speckle images $\boldsymbol{r_0}$ describes a stationary, pseudo-random process but does not contain independent, unbiased bits. Thus, $\boldsymbol{r_0}$ must be transformed through a randomness extraction procedure (sometimes called "digital whitening") from dim($\boldsymbol{r_0}$) pseudo-random bits to a shorter sequence of dim($\boldsymbol{k_0}$) ideally random, independent and unbiased bits. Several well-known methods of randomness extraction exist[36, 43, 44], along with more complex procedures such as seeded extractors[45]. While approaching the information-theoretic upper bound on efficiency in certain cases, all of these extraction methods are highly non-linear, and thus respond to errors caused by noise in an unpredictable manner. For example, a single erroneous flipped bit in an input to the extractors developed in ([36], [43], [44]) can alter the entire content and length of the whitened output, which is undesirable in our random key re-creation process that is known to contain a limited amount of noise (e.g., from laser fluctuations and sensor readout). Following, we present a linear procedure based on multiplication of two large matrices that both removes this limited noise and extracts randomness in as robust and accurate a way as possible. This procedure is diagrammed in Supplementary Figure 4.

We first describe our noise removal method applied before randomness extraction to an input speckle sequence. CPUF output noise corresponds to components of a speckle image that change gradually over time, which we observe to occur primarily within its larger spatial frequency components. Removing these components will allow an output key $\boldsymbol{k_0}(t = t_c)$ to more closely match a setup key $\boldsymbol{k_0}(t = t_0)$ created during public dictionary setup. Previous work[23, 26, 38] has also digitally altered detected speckle patterns to improve their functionality as robust identification keys. In these setups, a reflecting scatterer is inserted and removed from a reading terminal multiple times. Micron-scale misalignments of the scatterer lead to significantly different speckle images, from which similarities are extracted through a truncated discrete wavelet transformation (DWT). While the wavelet coefficients are unbiased, they remain significantly correlated, on top of requiring a very large data reduction factor (removing more than 99% of the original data). The DWT could also be applied to CPUF data to reduce noise before digital whitening (removing a much smaller fraction of high-frequency image coefficients). However, we find that a simple

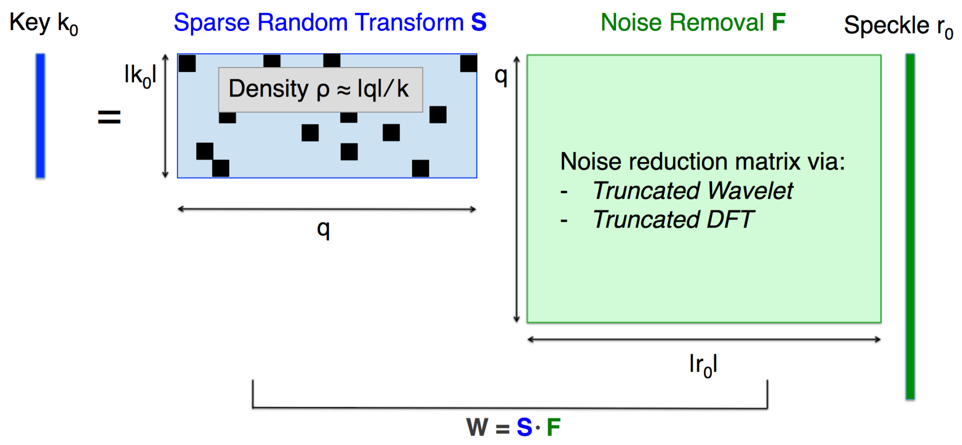

Supplementary Figure S4 | The digital whitening operator *W*. Diagram of the matrix ***W*** used to turn speckle input $\boldsymbol{r_0}$ into key $\boldsymbol{k_0}$. Typical sizes associated with the above operations are listed in Supplementary Table S1.

truncated discrete Fourier transform (DFT) removes noisy image content more efficiently than the DWT. The operation of either the truncated DWT or DFT may be expressed by a q x dim($\boldsymbol{r_0}$) rectangular matrix $\boldsymbol{F}$, where dim($\boldsymbol{r_0}$)/q is the operation's bit reduction factor. Since the CPUF's scatterer is fixed to the detector, noise over the course of the tested duration of 24 hours is quite minimal, often allowing for a very small reduction factor in the range dim($\boldsymbol{r_0}$)/$q \approx 0.9$-1.

After removal of high spatial frequencies, we adopt the linear randomness extraction operation recently suggested in ([29]) to turn the speckle vector $\boldsymbol{r_0}$ into a random key vector $\boldsymbol{k_0}$. This linear operation is performed as a sparse binary random matrix multiplication (assuming a binary basis). One large sparse random matrix $\boldsymbol{S}$ per device is constructed using a pseudo-random number generator and then saved digitally (approximately 10^{12} matrix entries, with 10^8 non-zero entries). We assume this matrix is publically known and may be accessed by an attacker without any loss of security. Its contained randomness adds no entropy to the random key $\boldsymbol{k_0}$. Instead, its randomized construction simply facilitates efficient entropy extraction from each biased, weakly random speckle vector.

In the absence of noise reduction, the number of columns in $\boldsymbol{S}$ matches the length of speckle vector $\boldsymbol{r_0}$, which for n_c concatenated images equals dim($\boldsymbol{r_0}$) $= n_c \cdot g = n_c \cdot g_x \cdot g_y$, where g_x and g_y are the number of detector pixels along the x and y dimensions, respectively, and again dim(·) indicates vector length. The noise reduction process shortens the speckle vector by a set bit reduction factor from dim($\boldsymbol{r_0}$) bits to q bits. The number of rows in $\boldsymbol{S}$ is set to the output key length dim($\boldsymbol{k_0}$) $= q/c$, $c > 1$, where c is a whitening reduction factor estimated by considering the amount of correlation based on speckle size and probe geometry, as well as the deviation of the random process from a uniform random process. Mathematically, q/c defines the size of the subspace that a speckle sequence is sparsely projected into. In practice, an example speckle data vector $\boldsymbol{r_0}$ from a particular CPUF configuration is processed as c is slowly increased until the output key $\boldsymbol{k_0}$ passes all Diehard and NIST randomness tests, indicating $\boldsymbol{k_0}$ is a sufficiently random OTP sequence for most applications of interest.

Parameter	Variable	Experimental Value
Sensor pixels (8-bit)	$g = g_x \cdot g_y$	$5.03\text{x } 10^6$
Raw speckle vector length	$\dim(r_0)$	$4.03 \text{ x } 10^7$
Noise removal size reduction	$\dim(r_0)/q$	0.95
Noise-removed speckle vector length	q	$3.83 \text{ x } 10^7$
Whitening reduction factor	c	25
Number of concatenated images	n_c	1
Output key vector length	$\dim(k_0) = n_c \cdot g/c$	$2.40 \text{ x } 10^6$
Sparse matrix density of 1's	ϱ	0.0001
Number of captured speckle images	n	5000
Error correction code rate	$1/\kappa$	0.025

Supplementary Table S1 | Experimental parameters. List of variables and their associated numerical values used for experimental CPUF demonstration.

After determining c, a second important parameter associated with matrix $\boldsymbol{S}$ is its measure of sparsity ϱ, which gives its density of non-zero matrix entries. An optimal value of ϱ is selected to balance desired whitening, output noise and memory capabilities. For highly biased sources, an ideal value of ϱ is 0.5. However, such a large density of ones both leads to a computationally challenging matrix operation for typical key sizes (~10^6 bits). Given an already pseudo-random speckle source, we find a much smaller density of $\varrho \approx 0.001$ is sufficient to generate keys that pass all statistical tests of randomness.

The noise minimization and randomness extraction matrices are applied together to create a key $\boldsymbol{k_0}$ from speckle vector $\boldsymbol{r_0}$ with $\boldsymbol{k_0} = \boldsymbol{SFr_0} = \boldsymbol{Wr_0}$, where $\boldsymbol{W}$ is the whitening operation described in the text (see Supplementary Figure S4). We note that this proposed randomness extraction method is efficient, but not optimal, for any r_0 following a Hidden Markov Process (HMP)[29]. As noted above, we guarantee r_0 follows an HMP using a Cauchy-Lorentz modulation mask at the scatterer's back surface, as discussed in ([27]). Variable values used in experiment (i.e., for the statistical tests and for the data in Fig. 4) are listed in Supplementary Table S1. Finally, we point out that while $\boldsymbol{W}$ must be "random" in the sense that each of the matrix elements of $\boldsymbol{S}$ should be uncorrelated, each device does not require a unique $\boldsymbol{W}$. The proposed CPUF protocol remains information-theoretically secure even if we assume the same matrix $\boldsymbol{W}$ is shared across every CPUF device and is publically known.

Supplementary Text D: Statistical randomness test performance

Statistical randomness test performance values for a typical sequence of concatenated random keys are presented both in Table 1 in the main text as well as Supplementary Table S2. Without a specific way to define ideal randomness for a finite sequence of bits, robust and thorough statistical testing is well recognized as the only possible method of effectively checking if a random number generation procedure is indeed effective[31, 46]. These statistical tests consist of establishing a null hypothesis of whether or not the tested sequence exhibits mathematical properties consistent with a random sequence, and then establishing within a given level of significance if the null hypothesis is verified.

Before testing, detected speckle data is whitened via the random matrix projection operator ***W*** explained above, using the same parameters as in the experimental demonstration (listed in Supplementary Table S1). The concatenated random vector containing all output keys passes all statistical tests (in both test packages) assuming a statistical significance level of α= 0.01. As described in the main text, typical results from the NIST randomness test associated with Special Publication 800-22[31,46] are in Table 1.

For the NIST suite, a tested 10^{10}-bit sequence of data is segmented into 10,000 sequences, each containing 10^6 bits, similar to the procedures used in prior randomness verification protocols[11,12] (our testing examines a much larger set of data). Note that all tests exhibit a passing p-value and proportional rate. In total, the NIST test was repeated on four different 10-gigabit data sequences for each of 3 unique CPUF devices. In between each of the four tests, significant heat was applied to the CPUF by setting its CMOS sensor to read-mode for several minutes, effectively "resetting" the device's key space. All independent test results followed similar statistics as those presented in Table 1 and Supplementary Table S2.

Supplementary Table S2 displays the results of applying the Diehard statistical test suite[30] to the *same* 10-gigabit data sequence as used for the NIST test results in Table 1. Since the Diehard test does not require segmentation of the data sequence, it was applied to the full 10-gigabit string of randomness. Again, we note that all tests exhibit a passing p-value. The Diehard test was also applied to 4 unique data sequences from 3 different CPUF devices, with similar passing statistics

**Statistical Test**	**p-value⁺**	**Pass/Fail**	**KS**
Birthday Spacings	0.816	Pass	Y
Overlapping Permutations	0.856	Pass	
Ranks of 31^2 Matrices	0.028	Pass	
Ranks of 32^2 Matrices	0.707	Pass	
Ranks of 6x8 Matrices	0.642	Pass	Y
Bitstream Test	0.051⁺ (18)	Pass	
Overlapping Pairs, Sparse Occupancy	0.099⁺ (22)	Pass	
Overlapping-Quadruples-Sparse-Occupancy	0.015⁺ (27)	Pass	
DNA	0.011⁺ (29)	Pass	
Count the 1`s	0.399	Pass	
Count the 1`s in Specific Bytes	0.008⁺ (25)	Pass	
Parking Lot Test	0.416	Pass	Y
Minimum Distance Test	0.530	Pass	Y
Random Spheres Test	0.090	Pass	Y
The Squeeze Test	0.812	Pass	
Overlapping Sums Test	0.002	Pass	Y
Runs Test	0.803	Pass	Y
Craps Test	0.130	Pass	

Supplementary Table S2 | Example Diehard statistical randomness tests performance. Diehard Random Test Suite performance for the same sequence of 10 gigabits of random CPUF data (concatenated into a single vector) as used in the NIST tests in Table 1. A p-value > 0.0001 indicates a significance level of α= 0.01, which is typically considered passing[11,12]. Tests using the Komolgorov-Smirnov (KS) test to obtain a single statistical p-value are denoted with a "Y" in the last column. The lowest p-value is displayed for tests that generate multiple p-values without using the KS test. These tests are denoted with a (⁺) after their p-value, followed by the number of tests used in parenthesis.

observed for each. The total length of concatenated data is limited to 10 gigabits due to computation time required primarily of the NIST test. Larger sequences of bits are expected to pass all tests until the device-specific total random bit upper bound *N* derived in Supplementary Text A is approached.

A third and final test applied to our experimentally generated 10-gigabit data sequences relies upon the context tree weighting (CTW) compression scheme, first developed in ([47]) and applied to estimate the randomness of PUF output in ([32]). We input an example 1-gigabit key sequence (after whitening with parameters in Supplementary Table S1) to an open-source version of the CTW algorithm, available from ([48]). We used the algorithm's default parameters and a tree-depth of 6. The algorithm outputs the length of the codeword required to compress the input 1-gigabit key, which was consistently slightly larger than 1-gigabit by approximately 10^5 bits. We assume this small fluctuation is a result of imperfections in the compression scheme, thus leading to our claim of an uncompressible random key with a per-bit entropy of exactly one in the main text.

Even after applying experimental and post-processing methods to reduce the fluctuation of speckle images over time, a limited amount of error remains between CPUF keys generated at dictionary setup time t_0 and at communication time t_c. The main cause of non-vanishing error lies in the whitening matrix $\boldsymbol{S}$, which must "mix" a pseudo-random speckle key with itself until it can pass all statistical tests. Specifically, this mixture consists of creating one bit within a final output key $\boldsymbol{k_0}$ from a modulo addition of $\varrho \cdot q$ random bits from the speckle vector $\boldsymbol{r_0}$. Here, $\varrho \cdot q$ equals the number of non-zero entries in one row of $\boldsymbol{S}$. The probability of an erroneous bit occurring is thus increased by a factor of $\varrho \cdot q$.

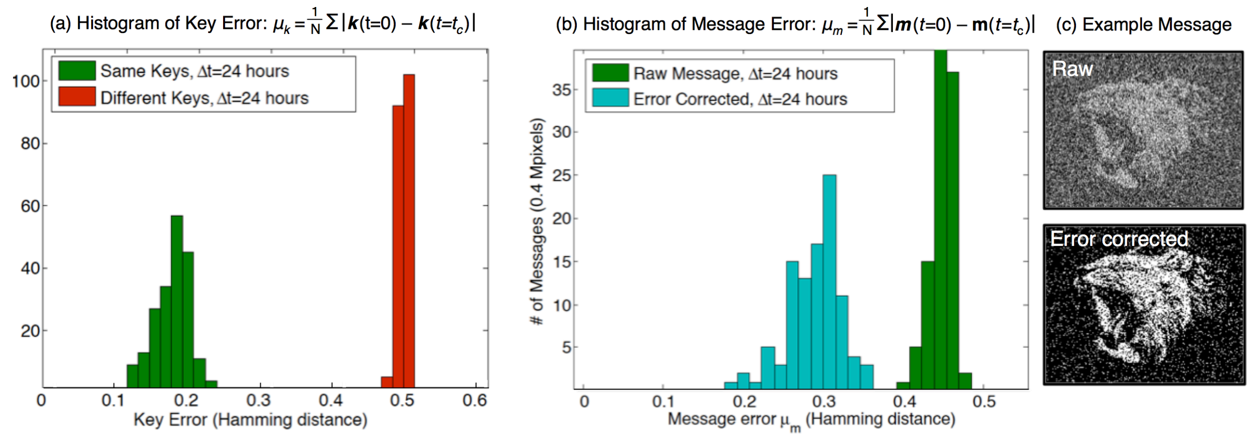

Supplementary Figure S5: Example experimental CPUF performance. (**a**) The normalized Hamming distance between 200 1Mbit CPUF keys created using 200 random SLM patterns at time *t=0*, and then re-created at a later time t_c=24 hours, is shown in green. When the same SLM pattern is used to re-create a key, only slight errors are observed after error correction (mean Hamming error at t_c=24 hours is $<\mu_k>$ = 0.17 bits). The red distribution displays the error between 100 1Mbit keys generated using *different* random SLM patterns, where $<\mu_k>$ = 0.50. This indicates that on average half the bits differ, as expected for keys that should be mutually random. (**b**) A similar histogram (green distribution) displays the Hamming error introduced to 200 messages after encryption and decryption (0.4Mbit length before and after error correction at t_c=24 hours). Performing both encryption and decryption mixes two keys per message, causing larger errors than in (a). Each message is encrypted and decrypted using a unique key-mixture. The blue distribution is the same histogram of message error after applying an error correcting procedure to minimize noise. Here, the mean error is reduced to $<\mu_m>$ = 0.29, and total transmittable bits are reduced by 9x. (**c**) Example messages demonstrate the effect of error correction.

Supplementary Text E: Error quantification and correction

As presented in the text, the effect of erroneous bits may be removed with error correction. Examined previously for applications in biometrics (i.e., fuzzy commitment and extraction) as well as authentication via integrated circuits, we refer the interested reader to references ([49-51, 33]) for details on security-preserving error correction with cryptographic applications. Since our demonstrated protocol is for message communication, we have the unique benefit of applying error correction directly to transmitted message bits. This construction is equivalent to the "code-offset construction" of fuzzy commitment detailed in ([48]), where the input $\boldsymbol{w}$ is simply the transmitted message, $\boldsymbol{m}$. In our experiment we utilized repetition coding, which is simply achieved by introducing redundancy into the transmitted message $\boldsymbol{m}$. For example, every bit of $\boldsymbol{m}$ can be repeated κ times to create $\boldsymbol{m}_r$, where $\dim(\boldsymbol{m}_r) = \kappa \cdot \dim(\boldsymbol{m})$. Encryption, message transmission and decryption of $\boldsymbol{m}_r$ unavoidably introduce bit-flip errors. For each segment of κ bits in the decrypted $\boldsymbol{m}_r$, corresponding to a single bit in the original message $\boldsymbol{m}$, we select the most frequent bit (the mode of the κ-bit set) as our estimate for the correct corresponding bit of the original message $\boldsymbol{m}$. This strategy requires that we use a key that is $\kappa \cdot \dim(\boldsymbol{m})$ bits to encrypt $\dim(\boldsymbol{m})$ bits of information, leading to a code rate of $1/\kappa$. Example experimental results of this process are in Supplementary Figure S5.

As noted in the main text, alternative tested error correction strategies included BCH codes and Hamming codes. These codes can improve the code rate and noise performance of message transmission at the price of additional computational complexity. One way to estimate the noise performance of the CPUF device, independent of a specifically selected error correction strategy, is to estimate the mutual information $I(X, Y)$ shared between a key set X generated at time t_0 and the same key set Y generated at time t_c. As shown in ([52]), $I(X, Y)$ sets an upper bound for the largest achievable "secrecy rate", or the highest percentage of bits within X and Y, that may be utilized for noise free communication. Following Section 6 of ([32]), it can be shown that $I(X, Y) = L(X) + L(Y) - L(XY)$, where $L(X)$ is the CTW-compressed codeword length of key set X. This formula was applied, along with the CTW algorithm with a tree depth of 6, to arrive at our secrecy rate upper-bound estimate of $I(X, Y) = 0.18$.

Finally, we note that our protocol maintains information-theoretic security with error correction given an ideally random key. However, we note that a non-ideal key, for example suffering from a bias away from a uniform distribution, may leak additional message information when error correction is applied[34]. More advanced error correction procedures using information reconciliation can also offer guaranteed security in such non-ideal cases, but require Alice and Bob to exchange several messages[53].

Supplementary Text F: Advanced security considerations and protocols

Several works offer a mathematical framework to describe the cryptographic behavior of a PUF. We refer the interested reader to ([54, 55, 19]) for a more detailed consideration of possible attacks on non-optical PUF's, and to ([24]) for a discussion regarding the difficulty of cloning, probing, or simulating an optical PUF's volumetric scattering material. Following, we consider the practical security of the CPUF's OTP-based protocol during typical use, and then turn to several cases of security given the CPUF is stolen by a malicious Eve.

1. Addressing practical concerns of OTP-based encryption

If one assumes that Alice and Bob's CPUF devices are never stolen, the proposed CPUF encryption scheme's security is effectively equivalent to that of a one-time-pad (OTP) based on a pseudorandom number source. It is direct to prove the OTP offers information-theoretic security[1]. Shannon's proof applies both to any encrypted message Alice sends as well as the key-mixtures saved within the public dictionary. Common attacks such as known-plaintext, chosen-plaintext, and chosen-ciphertext reveal no information about previous or future messages given an ideally operating CPUF. While this "perfect" theoretical security is a strong starting point for a new physical method of communication, OTP's still suffer from several specific attacks and downsides, even if we assume the keys remain absolutely hidden from any third party:

- *Bit-flipping attack*: The OTP is a malleable protocol[56]. An attacker may induce a predictable change in a message's plaintext m by altering its ciphertext c, without knowing the contents of m. Such attacks are prevented with an additional layer of security requiring message authentication, like a digital signature[57]. For example, if Alice encrypts m with its signature $S(m)$ appended to it, Bob can use $S(m)$ to validate whether m remained tamper-free during transmission. Including this digital signature thus allows Alice and Bob to ensure their CPUF-transmitted data is authentic.

- *Keystream re-use attack*: If a portion of any CPUF key is ever reused, the security of the CPUF protocol may be compromised. This concept is summarized by a keystream re-use attack (also called a replay attack), which is simply avoided by never re-using keys. If the CPUF's large space of randomness is not sufficient for a particularly large message, replacing the scatterer or heating it will effectively "reset" its space of randomness.

- *Re-synchronization attack*: As with any stream cipher, perfectly synchronizing the sender and receiver is a challenge, especially with the addition of random channel noise. For example, the insertion of one additional erroneous bit in Alice's encrypted message may "shift" a segment of the ciphertext with respect to Bob's key, thus leading to a partially incorrect decryption. Re-synchronization procedures can assist with this problem, but are susceptible to attack[58]. We note that the CPUF includes a natural synchronization function through its creation of a set of display pattern input – key output pairs. These pairs serve to discretize the OTP into shorter segments, which allow Alice and Bob to re-align their keys roughly every 10^6 bits, if necessary.

- *Required key size*: Ideal OTP's require their encryption key to contain as many bits as the transmitted message length[59]. We expect future CPUF's to be able to keep up with large data transmission demands. Given current scaling trends of CMOS sensors and LCD-based displays, it is feasible to expect CPUF devices to scale to multiple-terabit randomness generation in the near future (see the derivation Supplementary Text A). Ideal security may be sacrificed by switching CPUF operation to a public-key protocol, which may require less than 10^5 bits per communication session key. Such alternative protocols are discussed in detail in Section 4 below.

- *Bit destruction*: OTP users must be able to destroy the key bits they have already used. Digital storage mechanisms are inherently difficult to completely erase[3, 60]. Since the CPUF's keys rely upon multiple coherent scattering, any small scatterer perturbation will ripple through to significantly alter all keys, thus making them easily "erasable". Perturbations include moving or removing the sensor, heating or breaking the scatterer or generally inducing any irreversible change to the optical path. Such perturbations will

destroy all of Alice and Bob's key bits – the CPUF system does not allow partial key erasure.

Next, we examine the security claims that arise when a CPUF device is stolen, which helps set the CPUF's physical memory apart from storing communication keys within digital memory (e.g., Alice and Bob instead each hold a USB key with many random bits, which does not offer the security inherent to volumetric random bit storage).

2. Eve steals a CPUF, simple protocol

Before turning to specific claims, it is first useful to define what type of theft our CPUF protocol must protect against. As noted above, since it is currently impossible to accurately reproduce a disordered 3D volume at optical wavelength-scale resolution, Eve will *not* be able to simply replace Alice or Bob's CPUF with a replica. By periodically verifying the authenticity of the devices they hold through probing with randomly selected SLM patterns $\boldsymbol{p}$, Alice and Bob can effectively guard against such a "replica switching" attack. Thus instead, the CPUF protocol must defend against adversaries who attempt to temporarily remove a device for quick characterization. The protocol's security is then in effect measured in relation to *how long* it would take Eve to extract any useful information from a stolen device. In its current form, the simple CPUF protocol has a functional lifetime of approximately 50 hours, as noted in the main text and detailed below.

With a temporarily stolen device and control over the SLM display, Eve can attempt to quickly determine the scatterer's structure by recording its optical response to all orthogonal display patterns. This is equivalent to mathematically cloning the transmission matrix $\boldsymbol{T}$, which is of interest in scattering and time-reversal experiments[61]. In the simple form of the CPUF protocol, by characterizing and digitally storing $\boldsymbol{T}$, Eve can later eavesdrop and perform a man-in-the-middle attack by monitoring the SLM pattern $\boldsymbol{p}$ associated with each transmitted ciphertext. If Alice and Bob's database is fully populated with every SLM pattern leading to an independent key-mixture, then such an attack will require Eve to measure all elements of $\boldsymbol{T}$, which we claim requires an infeasible amount of time. As described in Supplementary Text A, $\boldsymbol{T}$ contains ~10^8 rows, while the SLM contains 2 x 10^6 pixels for the current CPUF device.

We will begin with the assumption that full characterization of $\boldsymbol{T}$ is possible by probing the scatterer by turning one SLM pixel "on" at a time, as performed in ([61]). This is a generous assumption since it assumes $\boldsymbol{T}$ may somehow be recovered from intensity-only measurements without detecting the complex field, which has yet to be demonstrated. Specifically, this assumption reduces the CPUF scattering inversion process to a linear problem. As equation (1) includes a squaring operation due to the measurement of optical intensity, the problem is certainly not linear, but we will give the attacker the benefit of the doubt. We also assume image capture at 1 frame per second, which is currently shorter than the lower exposure time limit of approximately 1.5 seconds required to prevent scatterer heating and speckle pattern decorrelation. Scatterer heating is caused by both a high illumination beam intensity as well as sensor heating during rapid readout. Decorrelation from a faster probe rate leads each recorded speckle pattern to change into a nearly uncorrelated pattern within minutes. Based upon these two modest assumptions, Eve will require 2 x 10^6 seconds, or approximately 23 days, to completely measure an ideal stolen device's scattering matrix $\boldsymbol{T}$. Assuming Eve can attach some cooling mechanism to probe the device at the SLM's maximum 24 frames-per-second rate, CPUF characterization will still require roughly 1 day. While it is reasonable to assume that Alice or Bob will notice

their device missing over such a long period, we aim future work at extending this device lifetime.

If Alice and Bob's public dictionary is only partially populated with key-mixtures, then Eve may use the public dictionary to determine which SLM pattern subset $\boldsymbol{p}_{1..n}$ will be used for future communication. In this situation, mathematical cloning time is simply proportional to the number of dictionary entries, n. The cloning time of approximately 50 hours cited in the text is derived assuming a public dictionary populated by $n = 1.3 \times 10^5$ SLM patterns, the maximum number of uncorrelated keys extractable from the current CPUF setup (see Supplementary Text A). For the experimentally demonstrated $n = 5000$ SLM pattern public dictionary, the cloning time is slightly over 2 hours. For such a small n, it may be beneficial for Alice and Bob to add a certain amount of extra random "seed" data to the public dictionary to lengthen Eve's required cloning time. Alice and Bob could sift through this additional data by filtering out unsuccessful keys each communication attempt. Alternatively, Alice and Bob may adopt a slightly more complex procedure to make it difficult for Eve to directly take advantage of their dictionary-setup data, as detailed next. This additional layer of security will still allow Alice and Bob to efficiently communicate, but forces Eve to attempt all dictionary display patterns to characterize a stolen device. What's more, this additional layer will prevent an attacker from quickly decrypting any of Alice and Bob's previous communication that she may have saved (since Alice and Bob publically share which SLM patterns they use each round). However, it comes at the price of increasing the proposed protocol's complexity.

3. Eve steals a CPUF, two-layer protocol

It is clear that given access to the SLM patterns $\boldsymbol{p}$ that Alice and Bob share, Eve's task at breaking the CPUF protocol becomes slightly easier, especially if Alice and Bob do not utilize the full amount of randomness stored within each of their device's volumetric scatterers. As a simple conceptual starting point, let us assume that Alice and Bob adopt a protocol like the Advanced Encryption Standard (AES) to encrypt each pattern $\boldsymbol{p}_i$ in $\boldsymbol{p}_{1..n}$ before saving it in the public dictionary. Put simply, this is analogous to Alice and Bob each password-protecting their digitally saved SLM patterns. With a stolen device but without direct knowledge of the digital private key (i.e., a password) used to encrypt each SLM pattern, Eve will not know which patterns to use for quick CPUF characterization, or which were used to create the keys for previously transmitted messages. Thus, it is clear that a security advantage in terms of required characterization time is gained if Alice and Bob agree to use a digital layer of encryption to slow down any direct characterization attack. This concept of a second security layer follows a well-known trend within the cryptography community of implementing multiple independent factors of authentication to preserve security.

We formalize this concept with a slight alteration to the CPUF protocol described in the text. Similar to Alice and Bob using a password to protect $\boldsymbol{p}$, the security of a public-key cryptography primitive can help prevent Eve from quickly characterizing a device. Instead of Alice and Bob both publically sharing the SLM set $\boldsymbol{p}_{1..n}$, they instead each have a unique set of random binary patterns, $\boldsymbol{p}_{1..n}(A)$ and $\boldsymbol{p}_{1..n}(B)$, saved locally in digital electronic memory on their respective devices. We assume only *one* of these pattern sets is public given a stolen device. If Eve has access to *both* devices, then the advantage of this protocol is lost.

Before device calibration, Alice and Bob agree upon a publically known prime base g and common prime number h, following ideas from the Diffie-Hellman exchange procedure[62]. During dictionary setup, Alice and Bob will securely share their random binary patterns with each other,

without saving them to digital memory. At the ith step, Alice and Bob will each compute and display on their SLM's the binary map PK[$\boldsymbol{p}_i(A)$, $\boldsymbol{p}_i(B)$] = $(h^{pi(A)} \bmod g)^{pi(B)}$. This shared "private key" will now serve as the ith shared display pattern between Alice and Bob. However, unlike the previous CPUF protocol construction, this shared display pattern is *never* saved in a public dictionary (i.e., it is never saved in digital memory). Instead, only the "public key" sets $\boldsymbol{p}_{1..n}(A)$ and $\boldsymbol{p}_{1..n}(B)$ are saved locally in digital electronic memory on each device, and the private key must be re-computed by Alice and Bob at a later time.

For example, if Alice is now separate from Bob and would like to send him a message, she first selects an un-used pattern from her local digitally saved dictionary of public keys, $\boldsymbol{p}_i(A)$. She then computes and publically sends to Bob both $(h^{pi(A)} \bmod g)$, as well as the index i. Bob selects $\boldsymbol{p}_i(B)$ from his digitally saved dictionary and computes $(h^{pi(B)} \bmod g)$, which he then sends to Alice. He also computes PK[$\boldsymbol{p}_i(A)$, $\boldsymbol{p}_i(B)$] = $(h^{pi(A)} \bmod g)^{pi(B)}$ using the random sequence received from Alice as the base of the exponent. Finally, Alice computes PK[$\boldsymbol{p}_i(B)$, $\boldsymbol{p}_i(A)$] = $(h^{pi(B)} \bmod g)^{pi(A)}$ using the random sequence she received from Bob as the base of her exponent. Note that although the public keys in the exponents have been switched, the Diffie-Hellmann protocol guarantees that the two sequences that Alice and Bob each compute in the last step are equivalent (i.e., PK[$\boldsymbol{p}_i(A)$, $\boldsymbol{p}_i(B)$] = PK[$\boldsymbol{p}_i(B)$, $\boldsymbol{p}_i(A)$]) given an appropriately chosen h and g ([62]). Alice and Bob now each display the pattern PK[$\boldsymbol{p}_i(A)$, $\boldsymbol{p}_i(B)$] on their SLM, and carry out the remainder of the CPUF protocol as laid out in the main text. In essence, the two-layer protocol above simply protects a publically known SLM pattern $\boldsymbol{p}_i$ with a computationally secured cryptographic primitive (Diffie-Hellman exchange). Any attempt to break this primitive must overcome the computational challenges associated with the discrete logarithm problem, which are the basis for most modern cryptosystems today.

The primary benefits of protecting the SLM patterns with a public-key protocol are threefold. First, with a stolen device from Alice, Eve will have access to the entire public key set $\boldsymbol{p}_i(A)$, but will still not know which SLM patterns PK[$\boldsymbol{p}_i(A)$, $\boldsymbol{p}_i(B)$] were used during dictionary setup. Thus, she will not be able quickly extract the n CPUF keys she expects Alice and Bob to exchange in the future by only displaying n probe patterns. Second, Eve will not be able to monitor which SLM patterns Alice and Bob shared in the past, and thus will lose the ability to efficiently decrypt previously shared messages. And third, even if Eve is able to crack the public key protocol, she must still have a way to recognize a correctly decrypted pattern PK[$\boldsymbol{p}_i(A)$, $\boldsymbol{p}_i(B)$] from the set of all possible patterns. Since this shared key is random, the only way to check if her decryption is the correct pattern is to pass it through the device *and* see if the generated speckle matches a known ciphertext-plaintext combination of a message previously sent by Alice or Bob. This will clearly significantly reduce her characterization speed, even in the presence of an extremely weak public-key scheme.

As a result of these three safeguards, Eve is left with several options in her quest to quickly characterize Alice's stolen device. First, she can try to repeatedly ask Bob to complete the public-key protocol with her. However, this procedure can be easily limited through safeguards on Bob's end. For example, if Bob applies a short time limit (e.g., 1 second) on the speed at which he agrees to complete the public-key screen protocol, Eve's cloning time will be effectively doubled. Since no CPUF keys should ever be re-used (to ensure the OTP's security), Bob may also reject any request for a previously used SLM pattern, preventing Eve from ever acquiring the screen patterns from previous communications.

Second, Eve can attempt to computationally crack the Diffie-Hellman exchange used to protect *each* "private key" SLM pattern PK[$\boldsymbol{p}_i(A)$, $\boldsymbol{p}_i(B)$]. For only a few SLM patterns, this will take a

considerably long time (typically multiple days). Alternatively, Eve can attempt to guess which pattern to display, although the probability of a correct guess from a set of ~10^5 independent binary elements that comprise $\boldsymbol{p}_i$ is effectively zero. Finally, Eve can just attempt to fully characterize $\boldsymbol{T}$, which is all the useful random information that is contained in the stolen CPUF device, and then apply whatever computational tools necessary to determine Alice and Bob's communication after returning the stolen device. This returns us to the notion of a device's functional lifetime set by the cloning time of $\boldsymbol{T}$, which is 50 hours for the current device but can extend to 23 days for a similarly sized ideal device. In summary, the CPUF two-layer security protocol provides an added measure of computational protection to ensure that Eve can only extract useful information from encrypted CPUF communication *after* spending a significant amount of time to mathematically clone the scattering matrix (50 hours – 23 days for the current model).

4. CPUF public key protocol

As mentioned in the main text, a secret key can be setup between two CPUF devices without requiring the formation of a secure connection prior to communication. Instead of following our modified OTP protocol, one of several public key protocols may be adopted instead, such as that outlined in Section 3 above to protect the shared SLM pattern. While they do not offer the OTP's perfect security, public key protocols may be of more practical use in situations where an initial secure meeting is inconvenient. Again focusing on one of the most common public key protocols, the Diffie-Hellman exchange procedure, helps to clarify these points. For CPUF-based Diffie-Hellman exchange, Alice and Bob must first establish a connection over a public channel to agree upon a publically known prime base g and common prime number p. Second, Alice and Bob each generate one private key, k_A and k_B, with their respective CPUF devices. Third, without saving k_A in any permanent memory, Alice computes (p^{k_A} mod g) (with mod the modulo operation) and sends this computed public key to Bob. Bob creates, computes and sends a similar public key to Alice (using his private key k_B instead of k_A in the exponent). Any intermediate party may obtain the value of these two public keys. Forth, Bob uses the public key created by Alice to compute the exponential (p^{k_A} mod g)^{k_B}, while Alice similarly computes (p^{k_B} mod g)^{k_A} with the public key created by Bob. These two final computed numbers are equal modulo g, leading to a shared secret between Alice and Bob that is very difficult for an eavesdropper Eve to determine (she must overcome the discrete logarithm problem to determine either this secret, k_A, or k_B from anything transmitted over the public channel). Once a shared secret is established, Alice and Bob may rely upon a variety of well-known symmetric key algorithms (e.g., AES) to send and receive encrypted messages.

To avoid any reliance upon digital memory, it is clear that Alice and Bob must each re-generate their private CPUF keys k_A and k_B in the fourth step. During a practical communication setup, Alice and Bob will actually have to re-generate k_A and k_B multiple times. With the above public-key protocol, these private keys must remain *absolutely* noise free each time they are generated. Unlike the modified OTP protocol, three points allow absolute noise-free key generation to be quite achievable under a public-key framework. First, each public key k_A and k_B can be quite short – approximately 3000 bits is a sufficient length. Thus, heavy error correction procedures that reduce several million original noisy bits to several thousand noise-free bits may be adopted. Second, keys must remain free of noise for only a short period of time. While the OTP protocol requires keys to remain noise-free from the time of public dictionary setup, which may potentially extend to many days, a public-key protocol only requires noise-free keys throughout the duration of Alice and Bob's communication (typically on the order of several hours, at most). Third, if Alice and Bob communicate for a particularly long period of time and a key error does occur, a

simple "fix" exists. Once an error is detected, Alice and Bob may simply just restart the public-key protocol with a new set of uncorrelated private keys. While leading to a new set of security measures that we will not explore in detail here, these three points generally suggest that CPUF-based public keys are a directly realizable and quite powerful encryption option in systems that wish to tradeoff ideal OTP security for a less stringent setup requirement.

Supplementary Material References:

35. Goodman, J. *Speckle Phenomena in Optics* (Ben Roberts and Company, 2007).
36. Elias, P. The efficient construction of an unbiased random sequence. *Ann. of Math. Stat.* **43**(3), 865-870 (1972).
37. Cover, T. M. & Thomas, J. A. *Elements of Information Theory* (John Wiley and Sons, Inc., 1991).
38. Skoric, B. On the entropy of keys derived from laser speckle: statistical properties of Gabor-transformed speckle. *J. Opt. A: Pure Appl. Opt* **10**, (2008).
39. Vellekoop, I.M. *Controlling the Propagation of Light in Disordered Scattering Media*. PhD thesis, Univ. Twente (2008).
40. van Putten, E. *Disorder-Enhanced Imaging with Spatially Controlled Light*. PhD thesis, Univ. Twente (2011).
41. Feng, S., Kane, C., Lee, P.A., & Stone, D. Correlations and fluctuations of coherent wave transmission through disordered media. *Phys. Rev. Lett*. **61**(7), 834-837 (1988).
42. Freund, I., Rosenbluh, M. & Feng S. Memory effects in propagation of optical waves through disordered media. *Phys. Rev. Lett.* **61**, 2328–2331 (1988).
43. von Neumann, J. Various techniques used in connection with random digits. *Applied Math Series* **12**, 36–38 (1951).
44. Peres, Y. Iterating Von Neumann's procedure for extracting random bits. *Ann. Stat.* **20**(1), 590-597 (1992).
45. Nisan, N. & Zuckerman, D. Randomness is Linear in Space. *J. Comput. Syst. Sci.* **52**(1), 43-52 (1996).
46. Kim, S. J., Umeno, K. & Hasegawa, A. *Corrections of the NIST statistical test suite for randomness.* arXiv:nlin.CD/0401040v1 (2004).
47. Willems, F. M. J., Shtarkov, Y. M., & Tjalkens, T. J. The context-tree weighting method: basic properties. *IEEE Trans. Info. Theory* **41**(3), 653-664 (1995).
48. Franken , E., Peeters, M. & Willems, F. M. J. *Context Tree Weighting Algorithm*. <http://www.ele.tue.nl/ctw/> [Accessed November 11th, 2013] (2013).
49. Juels, A. & Wattenberg, M. A fuzzy commitment scheme. *6th ACM Conf. on Computer and Communications Security*, ACM Press, 28-36 (1999).
50. Dodis, Y., Reyzin, M. & Smith, A. Fuzzy extractors: How to generate strong keys from biometrics and other noisy data. *Proc. EUROCRYPT 2004 LNCS* **3027**, 523–540 (2004).
51. Dodis Y., and Smith, A. Correcting errors without leaking partial information. *Proc. ACM Symposium on Theory of Computing*, 654-663 (2005).
52. U. Maurer. Secret key agreement by public discussion from common information. *IEEE Trans. Info. Theory* IT-39, 733-742 (1993).
53. Brassard, G. & Salvail, L. Secret key reconciliation by public discussion. *Proc. EUROCRYPT Advances in Cryptology 1993*, 410-423 (1994).
54. Armknecht, F., Maes, R., Sadeghi, A., Standaert, F. & Wachsmann, C. Formal foundation for the security features of physical functions. *Proc. IEEE Symp. Security and Privacy*, 397-412 (2011).
55. Ruhrmair, U. et al., Modeling attacks of physical unclonable functions. *ACM Conf. Computer Comm. Security (CCS)*, 237-249 (2010).
56. Dolev, D., Dwork C. & Naor, M. Nonmalleable cryptography. *SIAM J. Computing* **30**(2), 391-437 (2000).
57. Katz, J. & Lindell, Y. *Introduction to Modern Cryptography* Ch. 12 (Chapman & Hall/CRC Press, New York, 2007).
58. Daemen, J., Govaerts, R. & Vanderwalle, J. Resynchronization weaknesses in synchronous stream ciphers. *Proc. EUROCRYPT Advances in Cryptology 1993*, 159-167 (1994).
59. Schneier, B. *Applied Cryptography: Protocols, Algorithms, and Source Code in C, Second Edition* (John Wiley and Sons, New York, 1996)
60. Anderson, R. & Kuhn, M. Low cost attacks on tamper resistant devices. In *IWSP: International Workshop on Security Protocols*, 125-136 (1997).
61. Popoff, S., et al. Measuring the transmission matrix in optics: an approach to the study and control of light propagation in disordered media. *Phys. Rev. Lett.* **104**(10), 100601 (2010).
62. Diffie, W. & Hellman, M. New directions in cryptography. *IEEE Trans. Info. Theory* **22**(6), 644-654 (1976).
